# The Field of Science

**Published:** 1894-04-07

**Authors:** 


### The Field of Science.

It was lately decided by vote in the Boston Common Council that dissection of animals be strenuously forbidden in the public schools. This order was, however, subsequently modified to admit of the dissection of cold-blooded animals.

Parliament has again been presented with the annual report of the Medical Department of the Navy. Though somewhat bulky in form, as incorporating 100 pages of tabular matter, the report shows that the health of our Navy has been normal, if not satisfactory, during the period under review.

The "greatest" consultation in history has just been recorded through the medium of the Transatlantic Press. No less, it appears, than 423 physicians gathered round the bedside of a member of the Imperial family of China in her late illness. This mighty array of doctors, failing to discover the cause of the patient's indisposition, it remained for the intervention of a Buddhist priest to supply the deficiency. His explanation was ingenious, if not definitely logical. "Before there were railroads she was well. After there were railroads she was ill. What could be more clear than the conclusion he drew?"

We understand from a correspondent that in China it is not very unusual to have congregated for consultation in cases of Imperial sickness four or five hundred doctors. These are called in not so much for purposes of actual consultation as to increase the dignity of a ceremonial; they are but actors in a grotesque play of Imperial pomp. The Buddhist priest above alluded to is doubtless in Chinese philosophy the figure of mind, the representative professor "of wind and water" classified with Taouism by the common people. He controls, in their imaginations, the relations between these two elements, air (wind) and water, acting mediator between the spirits of these elements and believers. The common people of China believe that if they erect edifices, such as tombs, temples or houses in a wrong situation, that is, in "opposition" to the positive character of the elements wind and water of the locality, the owner of such edifices will be punished by the spirits with great calamity. It requires explanation for a more western mind to grasp the meaning of the spirit of the "opposition" used to this sense; but it seems clear enough to the Chinee. These superstitious beliefs are now the main obstacle to the building of railroads in the Empire. It has frequently been prophesied in China by this remarkable Professor of Wind and Water that the Imperial Family and that country generally will have some unseen fatality befall it because of the introduction of railroads.

The Public Health Act of 1891, dealing with the provision of proper mortuaries, post-mortem rooms, and accommodation for the holding of inquests, is gradually evincing a substantial working. Marylebone has provided a mortuary admirably designed for this purpose. Out of the forty sanitary districts in the Metropolitan area twelve only, so far, possess accommodation such as is designed by the statutory requirements. When the improved measures are duly put into force, the custom of holding coroner's inquests in public houses will be effectually remedied.

At the Congress of Hygiene, Dr. Korosi, of Buda Pesth, stated that the proportion of deaths among children from weakly constitutions, or maladies traceable to the mother, was twice as large among the children of mothers under twenty, as among the children of mothers over thirty. The author of this statement seemed pretty sure of his facts, since they were consequent upon a comparison of several thousand cases. His investigations on the subject showed further, that the healthiest offspring was born of mothers between twenty and thirty, united to husbands between thirty and forty. In Hungary 15 per cent. of the number of marriages shows that the brides are under twenty years of age, and in England 20 per cent.

An American contemporary, commenting on the evils of early marriages in Europe, which shows itself in the health of the children, declares that this evil does not exist in the same extent among American-born women. "The women of this country," as our contemporary puts it, "having been enfranchised, and being at liberty to-day to support themselves, even as their brothers may, it is but natural that this freedom should reflect itself in the marriage statistics. The statistics on this point which I find available," he continues. and which we now append, "are worthy of a special study."

Statistics of Marriages in New York City.

| | 1891. | 1892. |
|---|---|---|
| Average age of grooms (years) ... ... | 28.32 | 28.89 |
| Average age of brides (years) ... ... | 24.59 | 24.43 |
| Number of grooms under 20 years of age... | 120 | 145 |
| Number of brides under 20 years of age... | 2,839 | 2,959 |
| Age at which there was greatest number of grooms (years) ... | 25.29 | 25.29 |
| Age at which there was next greatest number of grooms (years) ... ... ... | 21.24 | 21.24 |
| Age at which there was greatest number of brides (years) ... ... ... ... | 21.24 | 21.24 |
| Age at which there was next greatest number of brides (years) ... ... ... | 25.29 | 25.29 |
| Total number of marriages ... | 15,764 | 16,001 |

